# Lasting Immunological Imprint of Primary Epstein-Barr Virus Infection With Associations to Chronic Low-Grade Inflammation and Fatigue

**DOI:** 10.3389/fimmu.2021.715102

**Published:** 2021-12-20

**Authors:** Børre Fevang, Vegard Bruun Bratholm Wyller, Tom Eirik Mollnes, Maria Pedersen, Tarjei Tørre Asprusten, Annika Michelsen, Thor Ueland, Kari Otterdal

**Affiliations:** ^1^ Section of Clinical Immunology and Infectious Diseases, Oslo University Hospital Rikshospitalet, Oslo, Norway; ^2^ Research Institute of Internal Medicine, Oslo University Hospital Rikshospitalet, Oslo, Norway; ^3^ Department of Pediatrics and Adolescent Health, Akershus University Hospital, Lørenskog, Norway; ^4^ Institute of Clinical Medicine, University of Oslo, Oslo, Norway; ^5^ Department of Immunology, Oslo University Hospital and University of Oslo, Oslo, Norway; ^6^ Research Laboratory, Nordland Hospital, Bodø and Faculty of Health Sciences, Kristian Gerhard Jebsen - Senter for Tromboseforskning (TREC), University of Tromsø, Tromsø, Norway; ^7^ Centre of Molecular Inflammation Research, and Department of Clinical and Molecular Medicine, Norwegian University of Science and Technology, Trondheim, Norway

**Keywords:** Epstein-Barr virus, EBV, infectious mononucleosis, fatigue, chronic fatigue syndrome, CFS, RANTES, CRP

## Abstract

**Background:**

Epstein-Barr virus (EBV) causes infectious mononucleosis (IM) that can lead to chronic fatigue syndrome. The CEBA-project (Chronic fatigue following acute EBV infection in Adolescents) has followed 200 patients with IM and here we present an immunological profiling of adolescents with IM related to clinical characteristics.

**Methods:**

Patients were sampled within 6 weeks of debut of symptoms and after 6 months. Peripheral blood mononuclear cells (PBMC) were cultured and stimulated *in vitro* (n=68), and supernatants analyzed for cytokine release. Plasma was analyzed for inflammatory markers (n=200). The Chalder Fatigue Questionnaire diagnosed patients with and without chronic fatigue at 6 months (CF+ and CF- group, respectively) (n=32 and n=91, *in vitro* and plasma cohorts, respectively.

**Results:**

Broad activation of PBMC at baseline, with high levels of RANTES (Regulated on activation, normal T-cell expressed and secreted) in the CF+ group, and broad inflammatory response in plasma with high levels of T-cell markers was obeserved. At 6 months, there was an increased β-agonist response and RANTES was still elevated in cultures from the CF+ group. Plasma showed decrease of inflammatory markers except for CRP which was consistently elevated in the CF+ group.

**Conclusion:**

Patients developing chronic fatigue after IM have signs of T-cell activation and low-grade chronic inflammation at baseline and after 6 months.

**Clinical Trial Registration:**

https://clinicaltrials.gov/, identifier NCT02335437.

## Introduction

Epstein-Barr virus (EBV) is a gamma-herpes virus causing acute infection and life-long latency in over 90% of the population (1). The acute infection has a wide range of presentations, from asymptomatic to life-threatening multi-organ involvement, with infectious mononucleosis (IM) with fever, pharyngitis, malaise and lymphadenopathy as a common manifestation in adolescence (2). The virus is transmitted through saliva, and whereas the virus enters the body through oropharyngeal epithelial cells it will rapidly infect peripheral B-cells for massive viral replication (2). This results in a powerful immune response from cytotoxic CD8+ T-cells but also NK-cells that are crucial for controlling the infection, and it is now believed that this immune activation rather than the viral infection cause the majority of signs and symptoms (3, 4).

The heterogeneous nature of EBV infection is reflected within the IM group, where severity and length of symptoms and viral replication varies considerably. Fatigue is a particularly prominent feature of acute EBV infection and can last for months and even develop into Chronic Fatigue Syndrome (CFS) (5). The causes of acute as well as long-lasting fatigue in EBV infection are unclear, but the strong and sustained immune response suggests that immunological mechanisms might be involved.

In support of this hypothesis, subtle immune alterations have been reported in numerous CFS studies; the most consistent finding appears to be a low-grade systemic inflammation, as reflected in elevated serum C-reactive protein (CRP) (6–8), elevated pro-inflammatory cytokines (9, 10) and increased levels of innate immunity gene products (11). Although a recent review failed to provide evidence for elevation of pro-inflammatory cytokines in CFS (12), low-grade inflammation has been hypothesized as a common pathophysiological phenomenon across fatigue states in general (13).

The CEBA-project (Chronic fatigue following acute EBV infection in Adolescents) has followed 200 patients with IM meticulously recording clinical and immunological characteristics. Previous studies from the CEBA-project have shown that high CRP level and T-cell count serve as baseline predictors of fatigue development (7) as well as characteristics of the chronic fatigue state 6 months after EBV infection (8).

Primary EBV infection is characterized by elevated serum levels of pro- and anti-inflammatory cytokines like interferon (IFN)-γ, tumor necrosis factor (TNF), tumor growth factor (TGF)-β and interleukin (IL)-10, while transcriptomic analyses of peripheral blood mononuclear cells (PBMC) in primary EBV-infection have shown a distinct genetic expression profile associated with hyperinflammatory syndromes (14). The consequences of these acute inflammatory changes on functional properties of immune cells are not clear and could be associated to important clinical features.

In this study from the CEBA-project, we wanted to do a functional immunological profiling of adolescents with IM at baseline and after 6 months and relate it to fatigue caseness. By stimulating PBMC in cell cultures *in vitro* we could detect and explore the activation of monocytes, T- and/or B-cells during and after the acute EBV-infection. We hypothesized a) that EBV-infection would result in an immunological imprint detectable 6 months later, and b) that this imprint would differ according to fatigue state at 6 months.

## Methods

### Study Design

This study is a part of the CEBA-project (Chronic Fatigue following acute EBV Infection in Adolescents; ClinicalTrial ID: NCT02335437), embracing a prospective, cross-sectional and randomized controlled design with a total follow-up time of 21 months. A detailed description has been provided elsewhere (7). Here, only prospective results from the first six months are reported. The project has been approved by the Norwegian National Committee for Ethics in Medical research. All participants provided written informed consent before inclusion.

Inclusion of participants lasted from March 2015 until November 2016. EBV-infected individuals identified through collaborating medical laboratories and fulfilling the following criteria were eligible (7): (i) A serological pattern indicating acute EBV infection; (ii) Age 12-20 years; and (iii) Living in the Norwegian counties Oslo, Akershus or Buskerud. Exclusion criteria were (i) More than six weeks since debut of symptoms suggesting acute EBV infection; (ii) Chronic disease that needed regular use of medication; and (iii) Pregnancy. Patients were sampled at baseline (visit 1, V1) and after 6 months (visit 2, V2) (Ref 7). 200 patients and 70 healthy controls were included in the main CEBA study and used in the plasma analyses of this sub study (cohort presented in ref 7). A smaller group (Patients: V1, n=68; V2, n=67. Controls n=20) was randomly selected for the *in vitro* assays of this sub study. Demographic, serologic, immunologic and clinical characteristics of patients at baseline and healthy controls included in the *in vitro* assays are presented in **Table 1**.

**Table 1:** Demographic and clinical characteristics of EBV patients in the *in vitro* cohort at baseline.

	EBV CF+	EBV CF-	Healthy controls (HC)	ANOVA	*Post-Hoc*
n	32	36	20		
Male/female	4/28	16/20	6/14	0.016*	
Age, years	16.7 ± 1.5	16.7 ± 1.5	17.8 ± 1.8	0.016	CF+,CF-<HC
Days from symptoms	28.7 ± 5.9	32.0 ± 5.8		0.027**	
**Antibodies**					
EBV VCA IgM titer	120.1 ± 51.3	120.0 ± 51.5	1.4 ± 0.4	<0.001	CF+,CF->HC
EBV VCA IgG titer	92.3 ± 80.6	78.8 ± 34.2	81.9 ± 89.2	ns	
EBNA IgG titer	1.0 ± 4.9	1.53 ± 6.2	175.3 ± 228	<0.001	CF+,CF-<HC
**Microbiology**					
Blood PCR copies	1315 ± 1742	878 ± 933	199 ± 274	0.008	CF+,CF->HC
Throat swab PCR +/-	26/3	32/2	5/15	<0.001*	
**Lymphocytes and subsets**					
Lymphocytes, 10^9^ cells/L	2.50 ± 1.0	2.32 ± 0.7	1.79 ± 0.4	0.006	CF+,CF->HC
CD4+, 10^6^ cells/L	885 ± 282	799 ± 235	780 ± 194	ns	
CD8+, 10^6^ cells/L	1022 ± 585	893 ± 346	483 ± 146	<0.001	CF+,CF->HC
CD19, 10^6^ cells/L	181 ± 90	210 ± 106	233 ± 58	ns	
**B-cell subpopulations (%)**					
Naive	80.8 ± 7.3	81.5 ± 7.5	77.7 ± 7.6	ns	
Transitoric	4.9 ± 2.9	5.8 ± 3.4	2.5 ± 1.7	<0.001	CF+,CF->HC
IgM memory	8.4 ± 2.9	8.6 ± 4.7	10.9 ± 3.5	ns	
**T-cell subpopulations (%)**					
CD4+ naive	61.4 ± 10.6	60.7 ± 11.6	62.5 ± 10.0	ns	
CD4+ memory	47.8 ± 10.3	51.5 ± 11.8	52.2 ± 10.6	ns	
CD8+ naive	57.0 ± 12.9	60.0 ± 13.5	75.0 ± 15.2	<0.001	CF+,CF-<HC
CD8+ early eff mem	29.7 ± 12.0	26.8 ± 10.5	10.0 ± 3.3	<0.001	CF+,CF->HC
CD8+ late eff mem	7.18 ± 5.2	7.0 ± 5.5	12.7 ± 13.1	0.025	CF+,CF-<HC
**NK-cell function (%)**	25.8 ± 7.3	27.8 ± 7.7	23.4 ± 7.5	n.s	

Continuous data are given as mean ± SD. ns, not significant. *Chi square test. **T-test.

### Blood Samples

Peripheral blood was drawn from patients and serum and plasma processed and frozen as described elsewhere (7).

### *In Vitro* Stimulation of PBMC

PBMC were isolated from venous blood by Isopaque-Ficoll as described elsewhere (15) from adolescent with EBV-infection (V1 = 68, V2 = 67) and 20 healthy controls. PBMC was incubated in flat-bottomed 48-well trays (5 ∙ 10^5^ cells/well; Costar) in medium alone [RPMI-1640 with 2 mM L-glutamine and 25 mM HEPES buffer (PAA Laboratories, Pasching, Austria) supplemented with 10% FCS (PAA Laboratories)] representing unstimulated cells or stimulated for 20 hours with phytohemagglutinin (PHA, 90 µg/ml; Remel, Dartford Kent, UK), pokeweed mitogen (PWM, 20 µg/ml; Sigma, Saint Louis, Missouri, USA), isoprotenerol (20 µM, Calbiochem, Merck Darmstadt Germany), Pam3CysSerLys4 (Pam3CSK4, 20 ng/ml; InVivoGen, San Diego, CA, USA), Polyinosinic-polycytidylic acid-low molecular weight (Poly (I:C)-LMW, 5 µg/ml; InVivoGen), Lipopolysaccharide (LPS, 20 ng/ml; InVivoGen), orioribonucleotid (ORN R2336, 1 µM; MACS Miltenyi Biotec, Bergisch Gladbach, Germany), CpG oligodeoxynucleotides (CpG ODN 2006, 3 µM; InVivoGen) and PepTivator Epstein Barr Virus Epstein–Barr nuclear antigen 1 (PepTivator EBV EBNA-1, 0.6 nmol/ml; MACS Miltenyi Biotec). Overview is shown in **Table 2**. Supernatant were harvested and stored at -30°C for analysis.

**Table 2:** Stimulants in the *in vitro* assay.

Stimuli	Receptor	Target cell
US		All
PHA	TCR	T-cell
PWM	BCR	B-cell, (T-cell)
Isoproterenol	β-AR	T-cell
Pam3CSK4	TLR1/2	Monocyte, (B-cell)
Poly I:C-LMW	TLR3	T-cell
LPS	TLR4	Monocyte
ORN	TLR7	Monocyte, (B-cell)
CpG ODN	TLR9	B-cell
PepTivator EBV EBNA-1	TCR/BCR	T-cell, B-cell

β-AR, β-adrenergic receptor; BCR, B-cell receptor; CpG ODN, CpG oligodeoxynucleotides; LPS, Lipopolysaccharide; ORN, orioribonucleotid; Pam3CSK4, Pam3CysSerLys4; PepTivator EBV EBNA-1, PepTivator Epstein Barr Virus Epstein–Barr nuclear antigen-1; PHA, phytohemagglutinin; Poly I:C-LMW, Polyinosinic-polycytidylic acid-low molecular weight; PWM, pokeweed mitogen; TCR, T-cell receptor; TLR, Toll-like receptor; US, unstimulated.

### Immunoassays

Cell culture supernatants were analyzed using a 27-Plex Panel multiplex cytokine assay comprising IL-1β, IL-1 receptor antagonist (IL-1Ra), IL-2, IL-4, IL-5, IL-6, IL-7, IL-9, IL-10, IL-12 (p70), IL-13, IL-15, IL-17, IFN-γ, TNF, Regulated on activation, normal T cell expressed and secreted (RANTES), Granulocyte colony-stimulating factor (G-CSF), Granulocyte-macrophage colony-stimulating factor (GM-CSF), basic fibroblast growth factor (bFGF), Platelet-derived growth factor (PDGF)-BB, Vascular endothelial growth factor (VEGF) as well as the chemokines IL-8/CXCL8, eotaxin1/CCL11, interferon-γ-inducing protein 10 (IP-10/CXCL10), monocyte chemotactic protein-1 (MCP-1/CCL2), and macrophage inflammatory proteins 1α (MIP-1α/CCL3) and 1b (MIP-1β/CCL4) by a multiplex cytokine assay (Bio-Plex Human Cytokine 27-Plex Panel; Bio-Rad Laboratories Inc., Hercules, CA, USA). The following cytokines were below the detection limit of the assay and therefore not included in further analyses: IL-4, IL-5, IL-7, IL-9, IL-12, IL-13, IL-15, IL-17A, eotaxin 1, GM-CSF, bFGF and PDGF-BB.

Concentrations of MCP-1/CCL2, IP-10/CXCL10, RANTES/CCL5, TGF-β 1, IL-2Rα/CD25 and CRP were measured in plasma from the whole patient cohort (V1 = 200, V2 = 195) and from 70 healthy controls using enzyme immunoassays. MCP-1 and IP-10 assays were purchased from Peprotech (Rocky Hill, NJ, USA) and the other assays from R&D Systems (Minneapolis, MN, USA). The inter- and intra-assay coefficients of variation were <10% for all of the assays.

### Questionnaires and Definition of Chronic Fatigue

A questionnaire distributed to all participants included the Chalder Fatigue Questionnaire (CFQ) (16). CFQ consists of 11 items scored on four-point Likert scales. In the present study, chronic fatigue caseness at 6 months follow-up were defined as a CFQ total dichotomous score of 4 or higher (each item scored 0-0-1-1), in accordance with previous clinical studies (17). Patients with and without chronic fatigue were termed EBV CF+ and EBV CF-, respectively.

### Statistics

Baseline demographics in patients and controls were compared using appropriate parametric and non-parametric analysis depending on characteristic type (categorical or continuous) and distribution. For comparison of cytokine levels at a given time-point between groups (i.e. plasma cytokines or *in vitro* responses), multivariate ANOVA was performed on log transformed levels with Bonferroni-adjusted *post-hoc* tests if appropriate (i.e. >2 groups). For paired analysis (i.e. comparison of temporal profile of plasma cytokines according to CF status), repeated measures ANOVA was utilized and p-values for the group- and time-effect are reported as well as their interaction. P-values are two-sided and considered significant when < 0.05.

## Results

### Serologic and Virologic Characteristics of the *In Vitro* Cohort

At baseline, patients had higher levels of EBV VCA IgM and lower levels of EBNA IgG antibodies, as well as higher frequency of EBV PCR+ throat swabs and higher number of EBV copies in peripheral blood, as compared to healthy controls (**Table 1**). There were no significant differences between the EBV CF+ and EBV CF- group.

### *In Vitro* Stimulation of PBMC at Baseline

**Figure 1:**
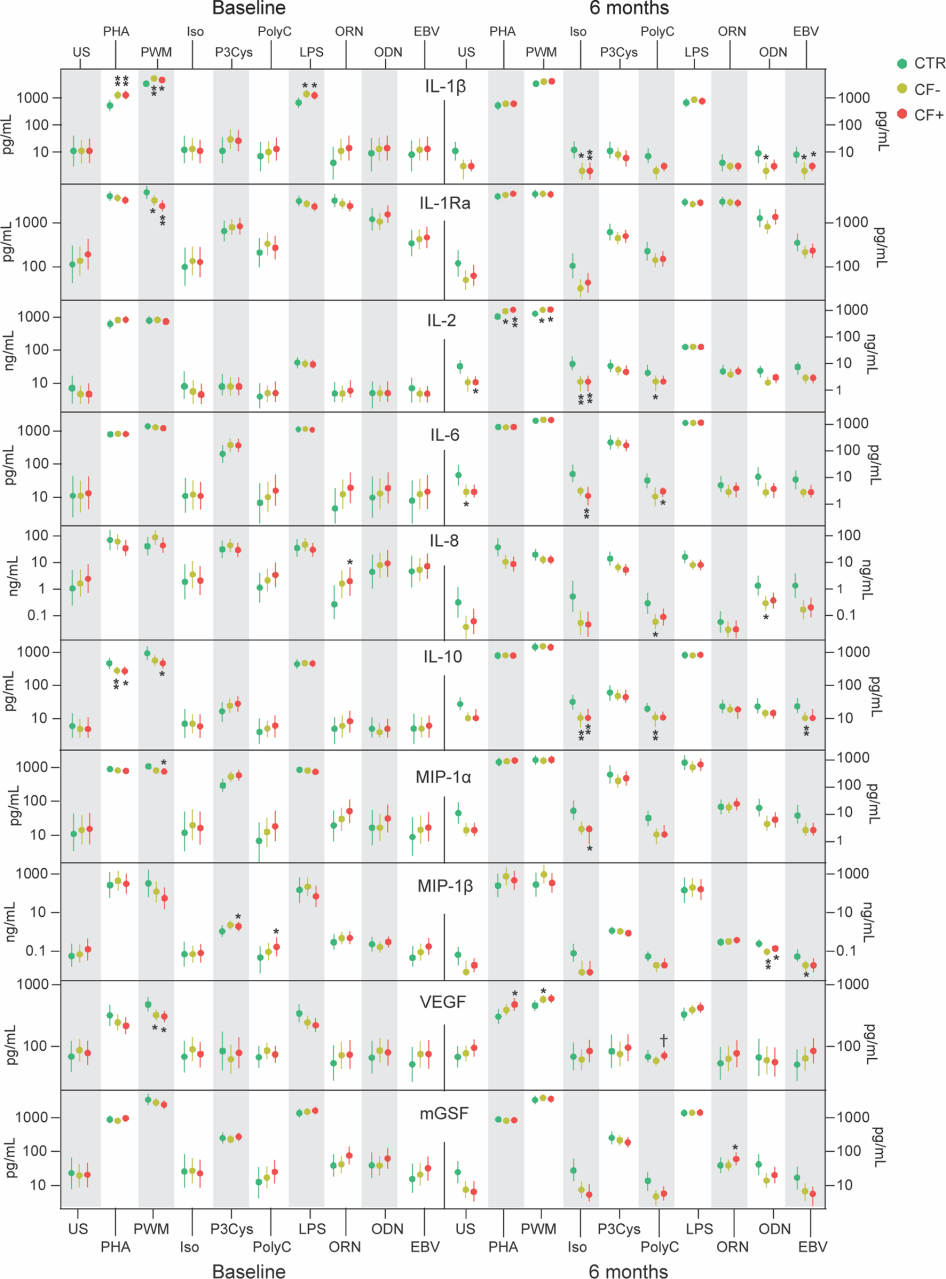
Supernatant levels of IL-1β, IL-1Ra, IL-2, IL-6, IL-8, IL-10, MIP-1α, MIP-1β, VEGF and mGSF in cell cultures of PBMC from healthy controls (CTR) and EBV-patients with and without chronic fatigue (CF+ and CF-, respectively) at baseline and after 6 months. *p<0.05, **p<0.01, CF+ and CF- vs HC. ^†^p<0.05, CF+ vs CF-.

**Figure 2:**
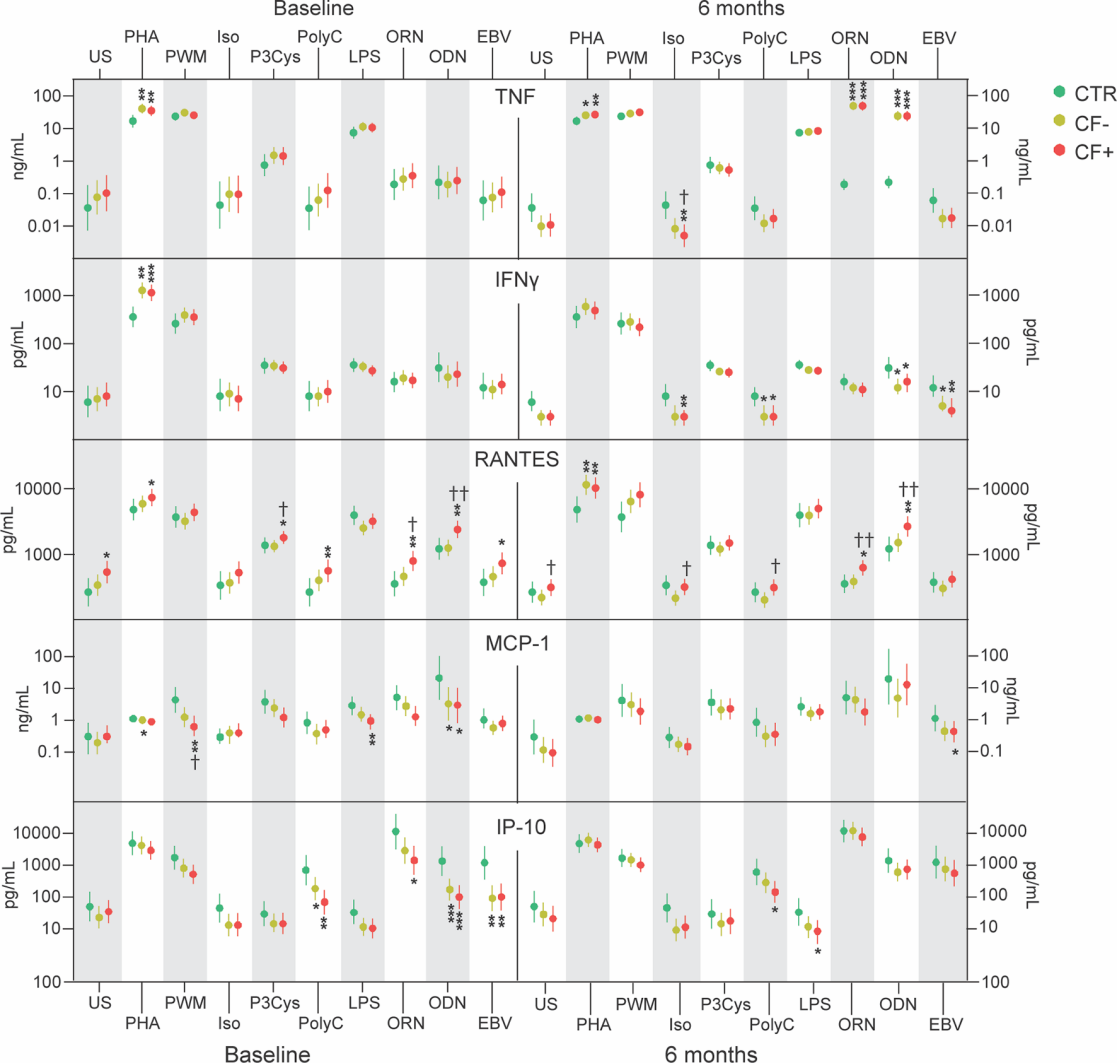
Supernatant levels of TNF, IFN- γ, RANTES, MCP-1 and IP-10 in cell cultures of PBMC from healthy controls (CTR) and EBV-patients with and without chronic fatigue (CF+ and CF-, respectively) at baseline and after 6 months. *p<0.05, **p<0.01, ***p<0.001 CF+ and CF- vs HC. ^†^p<0.05, ^††^p<0.01 CF+ vs CF-.

Cell cultures with PBMC from IM-patients and healthy controls were stimulated with agents targeting monocytes, T- and/or B-cells, and supernatants were analyzed for read-outs associated with the same cell populations (**Table 2**). Overall, these analyses showed clear activation of PBMC in patients as compared to healthy controls, but also significant differences between the EBV CF+ and EBV CF- group (**Figures 1**, **2** and **Supplemental Table 1**).

Several findings were notable when looking at differences between IM-patients and controls. Targeting monocytes with the TLR-4 ligand LPS we found higher levels of the upstream inflammatory mediator IL-1β in patients compared to controls (**Figure 1**). PHA binds to sugars on glycosylated surface proteins, including T-cell receptor (TCR), and thereby crosslinks them. PHA showed a strong response in secretion of TNF, IL-1β and the specific T-cell derived cytokine IFN-γ in cell cultures from patients as compared to controls (**Figures 1** and **2**). Interestingly, the important anti-inflammatory cytokine IL-10 was secreted in significantly lower levels in PBMC cultures from patients compared to controls when stimulated with PHA (**Figure 1**). Stimulating PBMC with the broad and unspecific mitogen PWM associated with B-cell activation we found increased levels of IL-1β but lower levels of IL-1RA and VEGF in patients as compared to controls (**Figure 1**). ODN is a ligand of TLR9 present in B-cells and showed a decreased response of MCP-1 and IP-10 in patients compared to healthy controls (**Figure 2**). EBV-antigen could theoretically stimulate both T- and B-cells and we observed a decrease in IP-10 secretion from patient cells compared to healthy control cells (**Figure 2**).

The two patient groups differed in response to several stimuli. ORN and Pam-3-Cys are ligands to the mainly monocyte expressed TLR7 and TLR1/2, respectively, and induced higher secretion of RANTES in the EBV CF+ group compared to both the EBV CF- group and healthy controls, discriminating between the two patient groups (**Figure 1**). T-cells were stimulated using the TLR3-ligand Poly I:C showing higher levels of RANTES in supernatants from EBV CF+ patients as compared to controls (**Figure 1**). There was an increased response of RANTES in the EBV CF+ group compared to both healthy controls and the EBV CF- group when stimulating with ODN (**Figure 2**). Stimulating with EBV-antigen there was an increase in RANTES secretion from EBV CF+ cells compared to healthy control cells (**Figure 2**).

Taken together, the *in vitro* stimulation assay at baseline showed a broad activation of PBMC in patients with particularly effect on levels of RANTES, TNF, IFN-γ and IL-1β. There was also a significant difference in RANTES levels between patient groups with higher levels in the EBV CF+ group.

### Plasma Cytokine Profile at Baseline

Plasma from patients with acute IM were analyzed for cytokines and inflammatory markers and compared to healthy controls. Overall, patients had a broad inflammatory response involving monocyte and T-cell associated markers (**Table 3**).

**Table 3:** Plasma cytokines and inflammatory markers at baseline.

	Controls	CF-	CF+	p-value
CRP, mg/L	0.66 (0.26, 1.22)	0.69 (0.25, 1.46)	1.04 (0.37, 3.37)*^††^	0.001
IP-10, pg/mL	29 (21, 48)	73 (48, 113)***	76 (53, 125)***	<0.001
MCP-1, pg/mL	132 (104, 172)	156 (116, 196)*	152 (124, 194)*	0.049
RANTES, ng/mL	1.33 (0.8, 2.05)	1.25 (0.65, 2.55)	1.4 (0.88, 2.83)	0.633
sTIM-3, ng/mL	5.92 (4.92, 7.32)	7.08 (5.92, 9.56)**	7.6 (5.8, 9.96)***	<0.001
sCD25, ng/mL	0.38 (0.26, 0.56)	0.5 (0.34, 0.85)*	0.48 (0.34, 0.83)**	0.016
TGF-β1, ng/L	2.06 (1.26, 2.6)	2.08 (1.52, 3)	2.38 (1.38, 3.11)	0.116

Data are shown as median and (25^th^, 75^th^) percentile. *p<0.05, **p<0.01, ***p<0.001 vs Controls. ^††^p<0.01 vs CF-.

The T-cell markers sCD25 and sTIM-3 were significantly elevated in plasma from IM-patients at baseline compared to controls, with no apparent difference between the EBV CF+ and the EBV CF- group. CRP showed significantly higher levels in the EBV CF+ than the EBV CF- group and healthy controls (**Table 3**). In contrast, RANTES was equally present in plasma from all patients and controls at baseline, but importantly platelets and not T-cells are the major cellular source of RANTES in plasma.

MCP-1 and IP-10 are considered markers of monocyte activation and there were significantly higher levels of both cytokines in patients compared to controls with no difference between the EBV CF+ and the EBV CF- group (**Table 3**).

### *In Vitro* Stimulation of PBMC at 6 Months

Cell cultures were repeated in all IM-patients after 6 months under the same condition as at baseline. Supernatant levels were compared to those of healthy controls at baseline. These analyses showed a long-lasting immunological imprint that was related to both the infection itself and symptoms of fatigue.

At 6 months, there were still notable differences between IM-patients and controls. The monocyte-associated ligand ORN targeting TLR7 showed raised levels of TNF secreted from both patient groups compared to healthy control cells (**Figure 2**). In contrast, Pam-3-Cys and LPS targeting TLR1/2 and TLR4, respectively, did not show any significantly different stimulatory effect between patient groups or healthy controls at 6 months, except from IP-10 that in response to LPS was secreted in significantly lower levels from EBV CF+ patients cells compared to control cells (**Figure 2**). Activation of T-cells with PHA showed consistently higher secretion of TNF, RANTES and IL-2 at 6 months for patients compared to controls, with no difference between the EBV CF+ and the EBV CF- group (**Figures 1** and **2**). TLR9-stimulation with ODN had a strong positive effect on the secretion of TNF at 6 months in patients compared to controls with no difference between the two patient groups (**Figure 2**). When stimulating the widely expressed beta-adrenergic receptor with isoproterenol, IM-patients showed lower levels of IL-1β, IL-2 and IL-10 compared to healthy controls (**Figure 1**)

EBV CF+ and EBV CF- patients had significantly different responses to a number of stimuli also at 6 months. Using ORN to stimulate TLR7, RANTES came out with significantly higher levels secreted from EBV CF+ cells compared to cells from the EBV CF- group and healthy controls (**Figure 2**). Stimulating PBMC with the T-cell associated TLR3-ligand Poly I:C, RANTES again seemed to discriminate between EBV CF- and EBV CF+ group, showing significantly higher levels in the latter group (**Figure 2**). The same was the case for VEGF upon Poly I:C stimulation (**Figure 1**). Further, there was a decrease in secretion of IFN- γ from both groups of patient cells compared to controls, while IL-2, IL-8 and IL-10 were lower in the EBV CF- group only (**Figures 1**, **2**). The EBV CF+ group was characterized by significantly lower levels of IL-6 and IP-10 compared to healthy controls when stimulating with Poly I:C (**Figures 1**, **2**). PHA induced higher secretion of VEGF from EBV CF+ cells compared to control cells, but with no difference between cells from the different patient groups (**Figure 2**).

Upon ODN stimulation, the secretion of RANTES was significantly higher in the EBV CF+ group compared to both healthy controls and the EBV CF- group (**Figure 2**). When stimulating the beta-adrenergic receptor with isoproterenol, the EBV CF+ group presented lower levels of TNF, IFN- γ, IL-6 and MIP-1 α than healthy controls (**Figures 1**, **2**). For TNF, there were significantly lower levels in the EBV CF+ group compared to the EBV CF- (**Figure 2**). RANTES was secreted in lower levels from EBV CF- cells compared to EBV CF+ cells, with no significant difference to cells from healthy controls (**Figure 2**).

Overall, at 6 months, TNF was shown to be elevated in the EBV CF-/CF+ group compared to healthy controls, while there were significantly higher levels of RANTES secreted from EBV CF+ cells compared to EBV CF- cells. Isoproterenol induced a decrease in secretion of cytokines from all patient cells, but in particular the EBV CF+ group.

### Plasma Cytokine Profile at 6 Months

**Figure 3:**
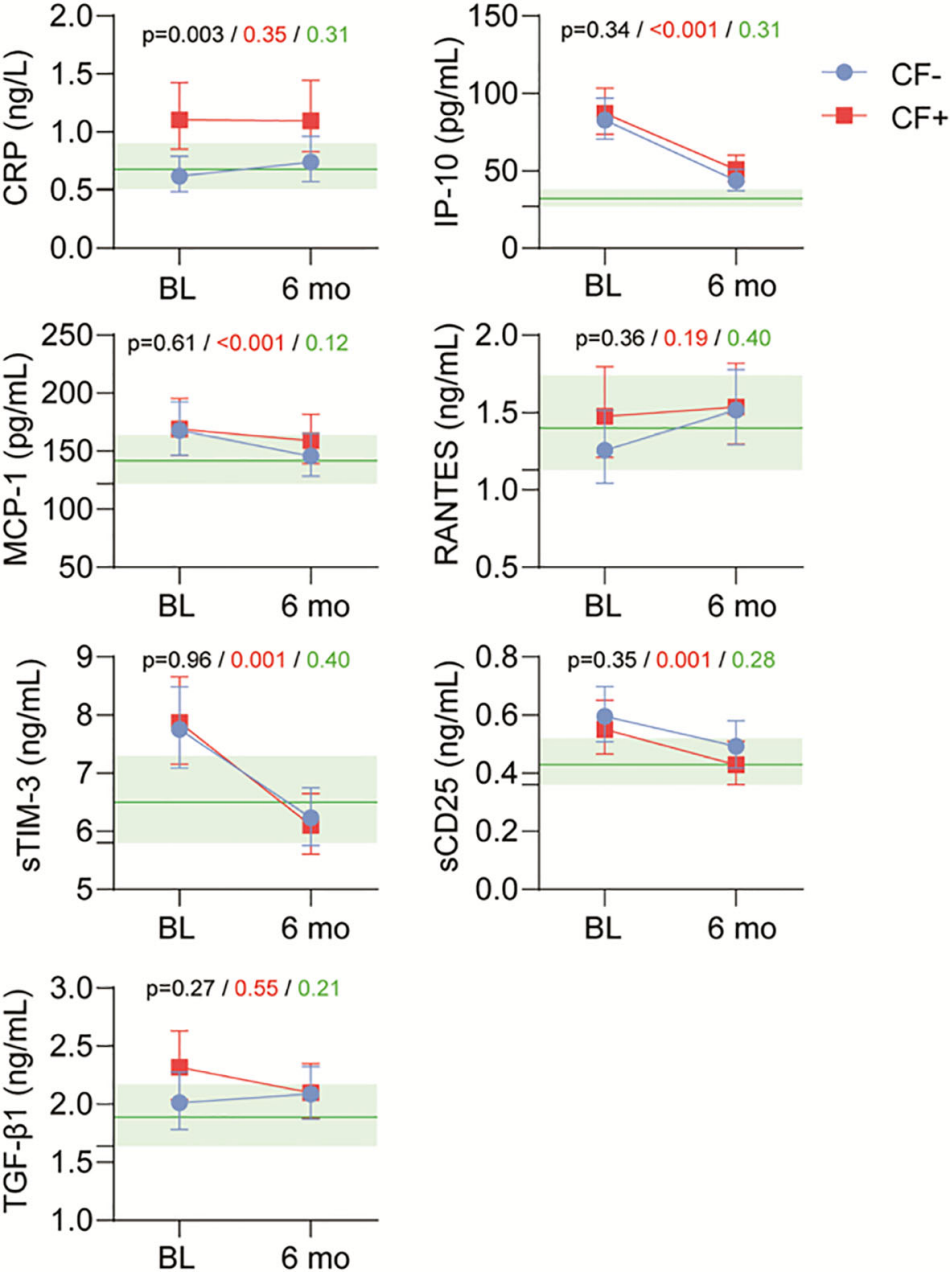
Plasma levels of cytokines and inflammatory markers in healthy controls (HC) and EBV patients with and without fatigue (CF+ and CF-, respectively) at baseline and after 6 months. Black p-value group effect, CF+ vs CF- irrespective of time. Red p-value time effect irrespective of group. Green p-value interaction of time vs group. Levels of controls with 95% CI marked in light green.

Analyzing cytokines in plasma, the monocyte markers IP-10 and MCP-1 as well as the T-cell markers sCD25 and sTIM-3 showed significantly lower levels in all patients after 6 months compared to baseline (**Figure 3**). There were no clear differences between patients in the EBV CF+ and EBV CF- group, but CRP was consistently elevated in EBV CF+ patients both at baseline and after 6 months.

### Comparing *In Vitro* and Plasma Samples

Cytokine levels in plasma and supernatants from cell cultures will both reflect immune activation and conditioning of immune cells. There was a significant correlation between plasma levels of the monocyte marker IP-10 and supernatant levels of IP-10 in patient cells when stimulated with Pam-3-Cys targeting monocyte expressed TLR1/2 (r=0.271, p=0.026). No such correlation was seen after 6 months or among healthy controls. There were no significant correlations between other monocyte or T-cell markers in the *in vivo* and *in vitro* samples (data not shown).

## Discussion

This study of acute infectious mononucleosis shows a broad and long-lasting inflammatory response in all patients, but with specific findings related to T-cell activation, low-grade inflammation and β-adrenergic response in patients developing chronic fatigue.

Firstly, the *in vitro* stimulation of PBMCs from IM-patients and healthy controls at baseline revealed signs of a clear T-cell response in all patients with increased supernatant levels of TNF, IFN-γ and RANTES to a range of stimuli. In plasma, the T-cell markers sCD25 and sTIM3 were similarly significantly raised in all patients. Repeating the *in vitro* stimulation after 6 months showed a continued increased T-cell response to PHA with elevated supernatant levels of TNF and IFN-γ, while none of the circulatory T-cell markers were elevated in the patient group after 6 months. Activation of T-cells, and in particular CD8+ T-cells, is an important factor in combating EBV-infection as shown by increased risk of EBV-associated pathologies in immunodeficiencies characterized by T-cell dysfunction (18). Clinically, IM is characterized by a long convalescence after the acute infection has subsided. The continued conditioning of T-cells from IM-patients observed here points to a corresponding long-lasting immunological imprint even if there were no signs of continued T-cell activation in plasma. Attenuated T-cell responses, possible related to immune exhaustion, has been implicated in the pathophysiology of chronic fatigue following infection (19). However, the results of the present paper do not support this hypothesis; rather the increased secretion of RANTES upon specific T-cell stimulation counts against an exhaustion paradigm.

Both *in vitro* and *in vivo* analyses showed signs of monocyte activation at baseline with increased response to monocyte stimulants like the TLR4-ligand LPS and the TLR7-ligand ORN, and raised levels of monocyte-associated markers like TNF and IL-1β, with a more blunted response after 6 months. Correspondingly, plasma levels of the monocyte markers IP-10 and MCP-1 were elevated in all patients at baseline but not after 6 months. Based on these findings, monocytes seem to exhibit a more contained response to EBV-infection than T-cells.

Secondly, even if the impact of the EBV-infection dominates the patient group as a whole, interesting findings were done in patients developing chronic fatigue (**Table 4**). The T-cell marker RANTES was significantly elevated in the EBV CF+ group compared to the EBV CF- group both at baseline and after 6 months, pointing to an early and persistent T-cell activation in these patients even if there were no difference in levels of the circulatory markers sTIM-3 and sCD25. In contrast, none of the monocyte markers *in vitro* or *in vivo* showed any discriminatory effect between patients with or without fatigue, suggesting T-cells rather than monocytes is associated to the development of fatigue.

**Table 4:** Summary of findings in the EBV CF+ patient group.

	Baseline	6 months
*In vitro*	↑ RANTES	↑ RANTES
		↑ β-agonist response
Plasma	↑ CRP	↑ CRP

β-adrenergic receptors are present in immune cells, including T-cells, and generally dampen immune responses *in vitro* (20). There seemed to be an increased response to the inhibitory effect of the β-agonist isoproterenol in the EBV CF+ group at 6 months with lower levels of both T-cell and monocyte associated cytokines. This could point to a dysregulation of adrenergic receptors or down-stream pathways in immune cells of IM-patients with fatigue. In contrast to signs of T-cell activation being present in the EBV CF+ group at an early stage, this increased β-adrenergic response seem to develop later. Interestingly, previous research has provided evidence of increased catecholamine levels as well as enhanced sympathetic nervous activity in chronic fatigue (6, 8, 21–23). Downregulation of the β-agonist receptor is a commonly observed phenomenon in hyperadrenergic states, but our findings do not seem to support a decrease in response in this patient group (24, 25).

CRP is a downstream inflammatory marker and mediator associated with activation of both T-cells and monocytes. Analyses of CRP in plasma in this study confirm previous findings of elevated levels in patients with chronic fatigue compared to not only healthy controls but also IM-patients without fatigue (7, 8).

Taken together, the results from the present study show that the general impact of EBV-infection on immune functions is more pronounced than the immunological differences associated with the development of chronic fatigue. In other words, there is a disproportional relationship between the substantial differences in symptoms and functions between the EBV CF+ and EBV CF- group, and the subtler immunological differences. Notably, baseline immunologic and serologic characteristics were indifferent between the two groups.

That said, we did observe statistical differences suggesting a slight inflammatory enhancement in the EBV CF+ group, corroborating previous reports (6–11). Interestingly, the differences were present already at baseline, indicating that low-grade inflammation is a risk factor for chronic fatigue after 6 months. The underlying mechanism for fatigue development, as well as the causes of the initial inflammatory enhancement, remain to be understood, and should be addressed in further research.

There are some limitations to our study, most notably related to the *in vivo* relevance of our *in vitro* findings. Even if experiments were done on a mixed cell population of PBMC we do not know the behavior of these cells in a natural setting. However, plasma levels of inflammatory markers seemed to correspond to our *in vitro* findings suggesting the experimental model to be of clinical relevance.

## Conclusion

In this sub study of the CEBA-project, analyses of *in vitro* and *in vivo* markers of inflammation and immune activation showed a broad response to acute EBV-infection. While most markers gradually normalized we could still detect significant signs of T-cell activation in all patients after 6 months. Patients developing chronic fatigue showed additional signs of T-cell activation and low-grade chronic inflammation at both baseline and after 6 months, as well as attenuated β-adrenergic response at 6 months. Levels of RANTES discriminated between patients with and without fatigue at an early stage of disease, and may represent an important focus point for further research efforts.

## Data Availability Statement

The raw data supporting the conclusions of this article will be made available by the authors, without undue reservation.

## Ethics Statement

The studies involving human participants were reviewed and approved by the South-Eastern Norway Regional Ethical Committee. Written informed consent to participate in this study was provided by the participants’ legal guardian/next of kin.

## Author Contributions

BF designed and performed research, analysed data, performed statistical analysis and wrote the article. VW designed and performed research, collected and analysed data, and co-wrote the article. TM designed and performed research, analysed data and co-wrote the article. MP performed research, collected data and co-wrote the article. TA performed research, collected data and co-wrote the article. AM performed research and co-wrote the article. TU designed and performed research, performed statistical analyses and co-wrote the article. KO designed and performed research, collected and analysed data, and co-wrote the article. All authors contributed to the article and approved the submitted version.

## Funding

The study was funded by South-Eastern Regional Health Authority in Norway [Grant number 2014007].

## Conflict of Interest

The authors declare that the research was conducted in the absence of any commercial or financial relationships that could be construed as a potential conflict of interest.

## Publisher’s Note

All claims expressed in this article are solely those of the authors and do not necessarily represent those of their affiliated organizations, or those of the publisher, the editors and the reviewers. Any product that may be evaluated in this article, or claim that may be made by its manufacturer, is not guaranteed or endorsed by the publisher.

## References

[1] Rostgaard K Balfour HH Jr Jarrett R Erikstrup C Pedersen O Ullum H. Primary Epstein-Barr Virus Infection With and Without Infectious Mononucleosis. PloS One (2019) 14:e0226436. doi: 10.1371/journal.pone.0226436 31846480 PMC6917282

[2] Dunmire SK Verghese PS Balfour HH Jr. Primary Epstein-Barr Virus Infection. J Clin Virol (2018) 102:84–92. doi: 10.1016/j.jcv.2018.03.001 29525635

[3] Meckiff BJ Ladell K McLaren JE Ryan GB Leese AM James EA. Primary EBV Infection Induces an Acute Wave of Activated Antigen-Specific Cytotoxic CD4(+) T Cells. J Immunol (2019) 203:1276–87. doi: 10.4049/jimmunol.1900377 PMC6697742 31308093

[4] Lam JKP Hui KF Ning RJ Xu XQ Chan KH Chiang AKS. Emergence of CD4+ and CD8+ Polyfunctional T Cell Responses Against Immunodominant Lytic and Latent EBV Antigens in Children With Primary EBV Infection. Front Microbiol (2018) 9:416. doi: 10.3389/fmicb.2018.00416 29599759 PMC5863510

[5] Hickie I Davenport T Wakefield D Vollmer-Conna U Cameron B Vernon SD. Post-Infective and Chronic Fatigue Syndromes Precipitated by Viral and non-Viral Pathogens: Prospective Cohort Study. Bmj (2006) 333:575. doi: 10.1136/bmj.38933.585764.AE 16950834 PMC1569956

[6] Sulheim D Fagermoen E Winger A Andersen AM Godang K Müller F. Disease Mechanisms and Clonidine Treatment in Adolescent Chronic Fatigue Syndrome: A Combined Cross-Sectional and Randomized Clinical Trial. JAMA Pediatr (2014) 168:351–60. doi: 10.1001/jamapediatrics.2013.4647 24493300

[7] Pedersen M Asprusten TT Godang K Leegaard TM Osnes LT Skovlund E. Predictors of Chronic Fatigue in Adolescents Six Months After Acute Epstein-Barr Virus Infection: A Prospective Cohort Study. Brain Behav Immun (2019) 75:94–100. doi: 10.1016/j.bbi.2018.09.023 30261303

[8] Kristiansen MS Stabursvik J O’Leary EC Pedersen M Asprusten TT Leegaard T. Clinical Symptoms and Markers of Disease Mechanisms in Adolescent Chronic Fatigue Following Epstein-Barr Virus Infection: An Exploratory Cross-Sectional Study. Brain Behav Immun (2019) 80:551–63. doi: 10.1016/j.bbi.2019.04.040 31039432

[9] Klimas NG Broderick G Fletcher MA. Biomarkers for Chronic Fatigue. Brain Behav Immun (2012) 26:1202–10. doi: 10.1016/j.bbi.2012.06.006 PMC5373648 22732129

[10] Montoya JG Holmes TH Anderson JN Maecker HT Rosenberg-Hasson Y Valencia IJ. Cytokine Signature Associated With Disease Severity in Chronic Fatigue Syndrome Patients. Proc Natl Acad Sci USA (2017) 114:E7150–e8. doi: 10.1073/pnas.1710519114 PMC5576836 28760971

[11] Nguyen CB Alsøe L Lindvall JM Sulheim D Fagermoen E Winger A. Whole Blood Gene Expression in Adolescent Chronic Fatigue Syndrome: An Exploratory Cross-Sectional Study Suggesting Altered B Cell Differentiation and Survival. J Transl Med (2017) 15:102. doi: 10.1186/s12967-017-1201-0 28494812 PMC5426002

[12] Blundell S Ray KK Buckland M White PD. Chronic Fatigue Syndrome and Circulating Cytokines: A Systematic Review. Brain Behav Immun (2015) 50:186–95. doi: 10.1016/j.bbi.2015.07.004 26148446

[13] Lacourt TE Vichaya EG Chiu GS Dantzer R Heijnen CJ. The High Costs of Low-Grade Inflammation: Persistent Fatigue as a Consequence of Reduced Cellular-Energy Availability and Non-Adaptive Energy Expenditure. Front Behav Neurosci (2018) 12:78. doi: 10.3389/fnbeh.2018.00078 29755330 PMC5932180

[14] Dunmire SK Odumade OA Porter JL Reyes-Genere J Schmeling DO Bilgic H. Primary EBV Infection Induces an Expression Profile Distinct From Other Viruses But Similar to Hemophagocytic Syndromes. PloS One (2014) 9:e85422. doi: 10.1371/journal.pone.0085422 24465555 PMC3894977

[15] Müller F Rollag H Gaudernack G Frøland SS. Impaired *In Vitro* Survival of Monocytes From Patients With HIV Infection. Clin Exp Immunol (1990) 81:25–30. doi: 10.1111/j.1365-2249.1990.tb05286.x 1974179 PMC1535002

[16] Chalder T Berelowitz G Pawlikowska T Watts L Wessely S Wright D. Development of a Fatigue Scale. J Psychosom Res (1993) 37:147–53. doi: 10.1016/0022-3999(93)90081-P 8463991

[17] White PD Goldsmith KA Johnson AL Potts L Walwyn R DeCesare JC. Comparison of Adaptive Pacing Therapy, Cognitive Behaviour Therapy, Graded Exercise Therapy, and Specialist Medical Care for Chronic Fatigue Syndrome (PACE): A Randomised Trial. Lancet (2011) 377:823–36. doi: 10.1016/S0140-6736(11)60096-2 PMC3065633 21334061

[18] Damania B Münz C. Immunodeficiencies That Predispose to Pathologies by Human Oncogenic γ-Herpesviruses. FEMS Microbiol Rev (2019) 43:181–92. doi: 10.1093/femsre/fuy044 PMC6435449 30649299

[19] Mandarano AH Maya J Giloteaux L Peterson DL Maynard M Gottschalk CG. Myalgic Encephalomyelitis/Chronic Fatigue Syndrome Patients Exhibit Altered T Cell Metabolism and Cytokine Associations. J Clin Invest (2020) 130:1491–505. doi: 10.1172/JCI132185 PMC7269566 31830003

[20] Padro CJ Sanders VM. Neuroendocrine Regulation of Inflammation. Semin Immunol (2014) 26:357–68. doi: 10.1016/j.smim.2014.01.003 PMC4116469 24486056

[21] Wyller VB Due R Saul JP Amlie JP Thaulow E. Usefulness of an Abnormal Cardiovascular Response During Low-Grade Head-Up Tilt-Test for Discriminating Adolescents With Chronic Fatigue From Healthy Controls. Am J Cardiol (2007) 99:997–1001. doi: 10.1016/j.amjcard.2006.10.067 17398200

[22] Wyller VB Godang K Mørkrid L Saul JP Thaulow E Walløe L. Abnormal Thermoregulatory Responses in Adolescents With Chronic Fatigue Syndrome: Relation to Clinical Symptoms. Pediatrics (2007) 120:e129–37. doi: 10.1542/peds.2006-2759 17606539

[23] Martínez-Martínez LA Mora T Vargas A Fuentes-Iniestra M Martínez-Lavín M. Sympathetic Nervous System Dysfunction in Fibromyalgia, Chronic Fatigue Syndrome, Irritable Bowel Syndrome, and Interstitial Cystitis: A Review of Case-Control Studies. J Clin Rheumatol (2014) 20:146–50. doi: 10.1097/RHU.0000000000000089 24662556

[24] Yu BH Dimsdale JE Mills PJ. Psychological States and Lymphocyte Beta-Adrenergic Receptor Responsiveness. Neuropsychopharmacology (1999) 21:147–52. doi: 10.1016/S0893-133X(98)00133-X 10379529

[25] Cases A Bono M Gaya J Jimenez W Calls J Esforzado N. Reversible Decrease of Surface Beta 2-Adrenoceptor Number and Response in Lymphocytes of Patients With Pheochromocytoma. Clin Exp Hypertens (1995) 17:537–49. doi: 10.3109/10641969509037423 7613527

