# Supplementary material for: Lasting Immunological Imprint of Primary Epstein-Barr Virus Infection With Associations to Chronic Low-Grade Inflammation and Fatigue
Source: Front Immunol. 2021 Dec 20;12:715102. doi: 10.3389/fimmu.2021.715102 (PMC8721200; doi:10.3389/fimmu.2021.715102)
Supplement: Supplementary file 1 [file DataSheet_1.pdf]

Table S1.Supernatant levels of cytokines in cell cultures of PBMC from healthy controls (HC) and EBV-patients (EBV) at baseline.

			Baseline 2 grp			
TNF	Stimuli	Receptor	Target cell	HC	EBV	*p*
	Unstim		All	37 (8 - 176)	88 (38 - 207)	0,330
	PHA	TCR	T-cell	17025 (11322 - 25602)	38392 (30772 - 47900)	0,001
	PWM		B-cell	23635 (18161 - 30758)	28166 (24416 - 32492)	0,248
	Isop.	β-AR	T-cell	44 (9 - 223)	95 (39 - 229)	0,410
	P3cys	TLR1/2	Monocyte, (B-cell)	753 (357 - 1591)	1462 (974 - 2192)	0,125
	Poly I:C	TLR3	T-cell	35 (8 - 157)	87 (38 - 196)	0,295
	LPS	TLR4	Monocyte	7442 (5064 - 10937)	11093 (9003 - 13668)	0,074
	ORN	TLR7	Monocyte, (B-cell)	190 (67 - 543)	313 (177 - 552)	0,410
	ODN	TLR9	B-cell	223 (70 - 709)	216 (115 - 404)	0,961
	EBV	TCR/BCR	T-cell, B-cell	62 (16 - 241)	90 (43 - 189)	0,628
IFN	Stimuli	Receptor	Target cell			
	Unstim		All	6 (3 - 13)	8 (5 - 11)	0,589
	PHA	TCR	T-cell	355 (223 - 564)	1200 (933 - 1544)	0,000
	PWM		B-cell	259 (165 - 408)	369 (289 - 472)	0,176
	Isop.	β-AR	T-cell	8 (4 - 18)	8 (5 - 12)	0,936
	P3cys	TLR1/2	Monocyte, (B-cell)	35 (24 - 49)	33 (27 - 40)	0,798
	Poly I:C	TLR3	T-cell	8 (4 - 16)	9 (6 - 13)	0,805
	LPS	TLR4	Monocyte	36 (26 - 48)	30 (26 - 35)	0,330
	ORN	TLR7	Monocyte, (B-cell)	16 (10 - 25)	18 (14 - 23)	0,618
	ODN	TLR9	B-cell	31 (16 - 63)	21 (15 - 31)	0,343
	EBV	TCR/BCR	T-cell, B-cell	12 (7 - 23)	12 (9 - 17)	0,935
IL-1β	Stimuli	Receptor	Target cell			
	Unstim		All	11 (3 - 37)	11 (6 - 21)	0,989
	PHA	TCR	T-cell	513 (334 - 789)	1253 (992 - 1581)	0,000
	PWM		B-cell	3268 (2696 - 3961)	4825 (4347 - 5355)	0,001
	Isop.	β-AR	T-cell	12 (4 - 38)	12 (6 - 22)	0,999
	P3cys	TLR1/2	Monocyte, (B-cell)	11 (4 - 34)	28 (15 - 50)	0,158
	Poly I:C	TLR3	T-cell	7 (2 - 23)	11 (6 - 21)	0,495
	LPS	TLR4	Monocyte	655 (438 - 979)	1292 (1039 - 1607)	0,004
	ORN	TLR7	Monocyte, (B-cell)	4 (1 - 15)	13 (7 - 25)	0,138
	ODN	TLR9	B-cell	9 (2 - 31)	13 (7 - 27)	0,557
	EBV	TCR/BCR	T-cell, B-cell	8 (2 - 26)	13 (7 - 25)	0,464
IL-1RA	Stimuli	Receptor	Target cell			
	Unstim		All	116 (49 - 277)	158 (98 - 253)	0,541
	PHA	TCR	T-cell	3989 (3234 - 4922)	3490 (3114 - 3911)	0,269
	PWM		B-cell	4473 (3309 - 6044)	2720 (2310 - 3203)	0,005
	Isop.	β-AR	T-cell	100 (42 - 238)	129 (81 - 207)	0,608
	P3cys	TLR1/2	Monocyte, (B-cell)	608 (381 - 971)	746 (579 - 962)	0,447
	Poly I:C	TLR3	T-cell	222 (114 - 432)	308 (215 - 442)	0,391
	LPS	TLR4	Monocyte	2923 (2332 - 3665)	2415 (2137 - 2730)	0,143
	ORN	TLR7	Monocyte, (B-cell)	3006 (2242 - 4030)	2455 (2094 - 2879)	0,231
	ODN	TLR9	B-cell	1249 (751 - 2077)	1321 (1003 - 1741)	0,847
	EBV	TCR/BCR	T-cell, B-cell	348 (192 - 630)	436 (316 - 602)	0,508
IL-2	Stimuli	Receptor	Target cell			
	Unstim		All	7 (3 - 14)	5 (3 - 8)	0,548
	PHA	TCR	T-cell	629 (485 - 816)	811 (705 - 935)	0,091
	PWM		B-cell	799 (642 - 994)	783 (696 - 882)	0,875
	Isop.	β-AR	T-cell	8 (4 - 18)	5 (4 - 8)	0,314
	P3cys	TLR1/2	Monocyte, (B-cell)	8 (5 - 16)	8 (6 - 11)	0,920
	Poly I:C	TLR3	T-cell	4 (2 - 9)	5 (3 - 7)	0,777
	LPS	TLR4	Monocyte	42 (30 - 57)	39 (32 - 46)	0,670
	ORN	TLR7	Monocyte, (B-cell)	5 (3 - 10)	6 (4 - 8)	0,757
	ODN	TLR9	B-cell	5 (2 - 10)	5 (3 - 7)	0,909
	EBV	TCR/BCR	T-cell, B-cell	7 (3 - 13)	5 (3 - 7)	0,385
IL-6	Stimuli	Receptor	Target cell			
	Unstim		All	11 (3 - 37)	12 (6 - 23)	0,887
	PHA	TCR	T-cell	811 (681 - 966)	828 (753 - 911)	0,838
	PWM		B-cell	1392 (1165 - 1663)	1283 (1165 - 1413)	0,427
	Isop.	β-AR	T-cell	11 (4 - 34)	12 (6 - 21)	0,969
	P3cys	TLR1/2	Monocyte, (B-cell)	211 (127 - 351)	361 (274 - 476)	0,068
	Poly I:C	TLR3	T-cell	7 (2 - 23)	12 (6 - 24)	0,384
	LPS	TLR4	Monocyte	1138 (928 - 1395)	1141 (1021 - 1274)	0,982
	ORN	TLR7	Monocyte, (B-cell)	5 (2 - 17)	14 (8 - 27)	0,142
	ODN	TLR9	B-cell	10 (3 - 36)	15 (8 - 29)	0,620
	EBV	TCR/BCR	T-cell, B-cell	8 (2 - 27)	13 (7 - 25)	0,479
IL-8	Stimuli	Receptor	Target cell			
	Unstim		All	1.1 (0.3 - 4.2)	1.9 (0.9 - 4)	0,461
	PHA	TCR	T-cell	69.4 (32.5 - 148.2)	48.1 (31.9 - 72.7)	0,403
	PWM		B-cell	43.2 (21.2 - 88)	63.6 (43.3 - 93.7)	0,344
	Isop.	β-AR	T-cell	1.9 (0.5 - 7.3)	2.7 (1.3 - 5.7)	0,632
	P3cys	TLR1/2	Monocyte, (B-cell)	31.9 (16.4 - 62.1)	36.6 (25.5 - 52.4)	0,722
	Poly I:C	TLR3	T-cell	1.2 (0.4 - 3.7)	2.5 (1.4 - 4.8)	0,239
	LPS	TLR4	Monocyte	36.3 (19.1 - 69.1)	38.8 (27.4 - 55)	0,856
	ORN	TLR7	Monocyte, (B-cell)	0.3 (0.1 - 1.3)	1.6 (0.8 - 3.3)	0,045
	ODN	TLR9	B-cell	4.6 (1.3 - 16.3)	8.2 (4.1 - 16.2)	0,427
	EBV	TCR/BCR	T-cell, B-cell	4.7 (1.5 - 15.1)	6 (3.2 - 11.4)	0,706
IL-10	Stimuli	Receptor	Target cell			
	Unstim		All	6 (3 - 13)	5 (3 - 8)	0,681
	PHA	TCR	T-cell	428 (313 - 586)	267 (225 - 316)	0,010
	PWM		B-cell	917 (618 - 1361)	539 (435 - 668)	0,021
	Isop.	β-AR	T-cell	7 (3 - 16)	6 (4 - 10)	0,764
	P3cys	TLR1/2	Monocyte, (B-cell)	17 (9 - 29)	25 (19 - 34)	0,197
	Poly I:C	TLR3	T-cell	4 (2 - 9)	5 (4 - 8)	0,593
	LPS	TLR4	Monocyte	446 (345 - 577)	468 (407 - 538)	0,748
	ORN	TLR7	Monocyte, (B-cell)	5 (2 - 10)	7 (5 - 11)	0,381
	ODN	TLR9	B-cell	5 (3 - 10)	5 (3 - 7)	0,654
	EBV	TCR/BCR	T-cell, B-cell	5 (2 - 12)	6 (4 - 9)	0,923

Table S1 (cont)

	Baseline 2 grp					
MCP-1	Stimuli	Receptor	Target cell	HC	EBV	*P*
	Unstim		All	283 (121 - 664)	248 (156 - 393)	0,784
	PHA	TCR	T-cell	1086 (924 - 1277)	919 (841 - 1003)	0,073
	PWM		B-cell	4042 (1837 - 8894)	1003 (654 - 1538)	0,003
	Isop.	β-AR	T-cell	275 (154 - 492)	390 (284 - 535)	0,298
	P3cys	TLR1/2	Monocyte, (B-cell)	3581 (1716 - 7473)	1807 (1213 - 2693)	0,108
	Poly I:C	TLR3	T-cell	817 (409 - 1633)	474 (325 - 690)	0,173
	LPS	TLR4	Monocyte	2653 (1506 - 4675)	1253 (922 - 1704)	0,023
	ORN	TLR7	Monocyte, (B-cell)	4860 (2189 - 10791)	2031 (1318 - 3131)	0,059
	ODN	TLR9	B-cell	19466 (4922 - 76990)	3548 (1683 - 7479)	0,033
	EBV	TCR/BCR	T-cell, B-cell	1044 (557 - 1957)	670 (476 - 942)	0,221
IP-10	Stimuli	Receptor	Target cell			
	Unstim		All	48 (19 - 121)	29 (17 - 47)	0,326
	PHA	TCR	T-cell	4827 (2321 - 10039)	3613 (2429 - 5374)	0,491
	PWM		B-cell	1637 (775 - 3458)	686 (457 - 1029)	0,045
	Isop.	β-AR	T-cell	42 (17 - 104)	14 (8 - 23)	0,038
	P3cys	TLR1/2	Monocyte, (B-cell)	28 (13 - 62)	15 (10 - 24)	0,175
	Poly I:C	TLR3	T-cell	569 (218 - 1485)	114 (68 - 193)	0,004
	LPS	TLR4	Monocyte	31 (15 - 68)	12 (8 - 17)	0,024
	ORN	TLR7	Monocyte, (B-cell)	11879 (3877 - 36397)	2571 (1401 - 4719)	0,019
	ODN	TLR9	B-cell	1348 (538 - 3377)	131 (80 - 216)	0,000
	EBV	TCR/BCR	T-cell, B-cell	1195 (419 - 3407)	95 (54 - 167)	0,000
MIP-1α	Stimuli	Receptor	Target cell			
	Unstim		All	11 (4 - 36)	14 (8 - 27)	0,721
	PHA	TCR	T-cell	892 (798 - 997)	810 (763 - 861)	0,136
	PWM		B-cell	1056 (903 - 1234)	795 (730 - 865)	0,002
	Isop.	β-AR	T-cell	12 (4 - 41)	18 (9 - 34)	0,608
	P3cys	TLR1/2	Monocyte, (B-cell)	305 (210 - 443)	540 (441 - 661)	0,009
	Poly I:C	TLR3	T-cell	7 (2 - 21)	14 (7 - 26)	0,277
	LPS	TLR4	Monocyte	839 (744 - 947)	780 (730 - 832)	0,289
	ORN	TLR7	Monocyte, (B-cell)	20 (8 - 48)	37 (23 - 59)	0,226
	ODN	TLR9	B-cell	17 (6 - 49)	22 (13 - 39)	0,658
	EBV	TCR/BCR	T-cell, B-cell	9 (3 - 29)	15 (8 - 28)	0,472
MIP-1β	Stimuli	Receptor	Target cell			
	Unstim		All	0.06 (0.02 - 0.2)	0.09 (0.05 - 0.19)	0,486
	PHA	TCR	T-cell	266 (74 - 952)	360 (180 - 718)	0,680
	PWM		B-cell	296 (73 - 1194)	91 (42 - 193)	0,143
	Isop.	β-AR	T-cell	0.07 (0.02 - 0.24)	0.07 (0.04 - 0.13)	0,947
	P3cys	TLR1/2	Monocyte, (B-cell)	1.14 (0.65 - 1.97)	2.02 (1.5 - 2.72)	0,072
	Poly I:C	TLR3	T-cell	0.05 (0.01 - 0.15)	0.12 (0.06 - 0.22)	0,171
	LPS	TLR4	Monocyte	145 (39 - 538)	125 (62 - 255)	0,843
	ORN	TLR7	Monocyte, (B-cell)	0.3 (0.14 - 0.65)	0.47 (0.31 - 0.71)	0,328
	ODN	TLR9	B-cell	0.23 (0.11 - 0.48)	0.22 (0.15 - 0.33)	0,911
	EBV	TCR/BCR	T-cell, B-cell	0.05 (0.02 - 0.16)	0.12 (0.07 - 0.21)	0,219
RANTES	Stimuli	Receptor	Target cell			
	Unstim		All	268 (166 - 435)	426 (328 - 554)	0,098
	PHA	TCR	T-cell	4815 (3367 - 6886)	6519 (5369 - 7915)	0,143
	PWM		B-cell	3707 (2577 - 5330)	3702 (3040 - 4508)	0,995
	Isop.	β-AR	T-cell	341 (212 - 548)	440 (340 - 569)	0,351
	P3cys	TLR1/2	Monocyte, (B-cell)	1378 (1055 - 1800)	1542 (1334 - 1782)	0,464
	Poly I:C	TLR3	T-cell	268 (166 - 432)	474 (366 - 614)	0,040
	LPS	TLR4	Monocyte	3949 (2860 - 5453)	2818 (2365 - 3357)	0,071
	ORN	TLR7	Monocyte, (B-cell)	360 (235 - 552)	600 (476 - 756)	0,040
	ODN	TLR9	B-cell	1219 (833 - 1785)	1705 (1386 - 2096)	0,128
	EBV	TCR/BCR	T-cell, B-cell	380 (238 - 605)	576 (448 - 742)	0,121
MGSF	Stimuli	Receptor	Target cell			
	Unstim		All	23 (9 - 58)	20 (12 - 33)	0,785
	PHA	TCR	T-cell	890 (705 - 1122)	885 (780 - 1003)	0,968
	PWM		B-cell	3377 (2465 - 4628)	2672 (2253 - 3170)	0,197
	Isop.	β-AR	T-cell	26 (10 - 73)	25 (14 - 43)	0,918
	P3cys	TLR1/2	Monocyte, (B-cell)	253 (186 - 345)	251 (212 - 296)	0,959
	Poly I:C	TLR3	T-cell	13 (5 - 31)	20 (13 - 32)	0,362
	LPS	TLR4	Monocyte	1383 (1083 - 1766)	1552 (1359 - 1772)	0,412
	ORN	TLR7	Monocyte, (B-cell)	40 (21 - 78)	55 (39 - 79)	0,402
	ODN	TLR9	B-cell	40 (19 - 86)	48 (32 - 73)	0,670
	EBV	TCR/BCR	T-cell, B-cell	16 (7 - 38)	25 (16 - 40)	0,370
VEGF	Stimuli	Receptor	Target cell			
	Unstim		All	62 (40 - 96)	72 (57 - 91)	0,562
	PHA	TCR	T-cell	246 (183 - 331)	190 (162 - 223)	0,131
	PWM		B-cell	354 (288 - 435)	254 (227 - 284)	0,006
	Isop.	β-AR	T-cell	62 (40 - 98)	72 (56 - 92)	0,566
	P3cys	TLR1/2	Monocyte, (B-cell)	74 (42 - 129)	64 (47 - 86)	0,640
	Poly I:C	TLR3	T-cell	61 (45 - 84)	70 (59 - 83)	0,466
	LPS	TLR4	Monocyte	262 (205 - 336)	194 (169 - 222)	0,037
	ORN	TLR7	Monocyte, (B-cell)	50 (30 - 83)	63 (48 - 84)	0,407
	ODN	TLR9	B-cell	60 (38 - 96)	72 (56 - 93)	0,510
	EBV	TCR/BCR	T-cell, B-cell	48 (29 - 79)	65 (49 - 86)	0,287
